# Facial pruritus and stiffness as the initial manifestations of glycine receptor antibody-positive progressive encephalomyelitis with rigidity and myoclonus: a case report and literature review

**DOI:** 10.3389/fimmu.2026.1955606

**Published:** 2026-10-09

**Authors:** Qiang Zhang, Jie Li, Jianguang Tang, Jia Fang

**Affiliations:** 1 Department of Neurology, Second Xiangya Hospital, Central South University, Changsha, China; 2 Clinical Medical Research Center for Stroke Prevention and Treatment of Hunan Province, Department of Neurology, Second Xiangya Hospital, Central South University, Changsha, China

**Keywords:** bulbar symptoms, facial pruritus, glycine receptor antibody, immunotherapy, misdiagnosis, progressive encephalomyelitis with rigidity and myoclonus, stiff-person spectrum disorders

## Abstract

Progressive encephalomyelitis with rigidity and myoclonus (PERM) is a rare and severe phenotype within stiff-person spectrum disorders (SPSD), frequently associated with glycine receptor antibodies (GlyR-Ab). Because typical rigidity and stimulus-sensitive spasms may be absent at disease onset, early diagnosis can be challenging. We report a 52-year-old man who presented with facial pruritus, facial stiffness, limited tongue protrusion, dysphagia, and left lower-limb pain. Early manifestations included diplopia, restricted extraocular movements, dysarthria, restricted mouth opening, involuntary jaw closure resembling trismus, and involuntary tongue biting. Nonspecific oropharyngeal and maxillofacial abnormalities on imaging initially raised concern for infectious multiple cranial neuropathy, tetanus, or orofacial infection; however, antimicrobial therapy and symptomatic treatment with diazepam and pregabalin produced no sustained improvement. The subsequent emergence of neck, truncal, and lower-limb stiffness, gait impairment, perioral hypersensitivity, and stimulus-induced facial muscle contractions prompted reconsideration of SPSD. Needle electromyography (EMG) demonstrated continuous synchronous motor unit activity in agonist and antagonist muscles at rest. Serum antibody testing was positive for glycine receptor 1 immunoglobulin G (GlyR1-IgG), with a reported semiquantitative value of 1:100, whereas serum glutamic acid decarboxylase 65 (GAD65) antibody was negative, supporting the diagnosis of GlyR-Ab-positive PERM. The patient showed marked initial improvement following combined immunotherapy and symptomatic treatment. During regular follow-up, the patient’s symptoms progressively improved. At the latest follow-up, diplopia, facial sensory abnormalities, restricted mouth opening, and limb rigidity had resolved. Repeat serum testing at a different laboratory showed persistent GlyR1-IgG positivity, with a reported endpoint titer of 1:100; the two numerical results were not directly comparable because different titration protocols were used. The patient was advised to continue oral mycophenolate mofetil 500 mg twice daily as maintenance therapy. This case illustrates the diagnostic challenges of GlyR-Ab-positive PERM when facial pruritus and cranial-bulbar symptoms precede generalized stiffness. Recognition of evolving stimulus-induced muscle contractions, together with characteristic EMG findings and antibody testing, can help distinguish this presentation from infectious cranial neuropathy, tetanus, and local orofacial disease.

## Introduction

Stiff-person syndrome (SPS) is the prototypical phenotype within the stiff-person spectrum disorders (SPSD), a group of rare immune-mediated neurological disorders characterized by progressive muscle rigidity, painful stimulus-sensitive spasms, and exaggerated startle responses (1). Progressive encephalomyelitis with rigidity and myoclonus (PERM) represents the severe end of SPSD and is distinguished from classic SPS by rapid progression, prominent myoclonus, hyperekplexia, brainstem involvement, long-tract signs, and autonomic dysfunction (1, 2).

SPSD is associated with antibodies targeting glutamic acid decarboxylase 65 (GAD65), the glycine receptor (GlyR), amphiphysin, dipeptidyl peptidase-like protein 6 (DPPX), gephyrin, and other proteins involved in inhibitory synaptic signaling (1, 3). Among these antibodies, glycine receptor antibodies (GlyR-Ab) are particularly associated with PERM and related SPSD phenotypes (2, 4). However, GlyR-Ab-positive PERM does not always present initially with the typical features of axial rigidity, painful spasms, and myoclonus; instead, early manifestations may be dominated by brainstem manifestations, dysesthesia, pruritus, or autonomic dysfunction (2, 5–7). These early presentations may mimic infectious cranial neuropathy, brainstem encephalitis, tetanus, orofacial infection, paraneoplastic brainstem syndromes, or functional neurological or psychiatric disorders, thereby delaying antibody testing and timely initiation of immunotherapy (2, 6–8).

Treatment of PERM relies primarily on symptomatic therapy and immunotherapy (1, 9). Here, we report a case of GlyR-Ab-positive PERM in which facial pruritus and cranial-bulbar and orofacial symptoms preceded axial and limb stiffness. We compare the clinical course with previously reported presentations, focusing on the diagnostic overlap with infectious cranial neuropathy, tetanus, and local orofacial disease.

## Case description

A 52-year-old man was admitted to our hospital on April 4, 2025, with a 20-day history of facial pruritus and stiffness, a 1-week history of limited tongue protrusion, involuntary jaw closure, and dysphagia, and a 1-day history of left lower-limb pain. The illness began with facial pruritus in the absence of facial pain, facial asymmetry, drooling, ptosis, or other accompanying symptoms. He subsequently developed cold-induced facial stiffness, left-sided biting difficulty, restricted mouth opening, diplopia, and head heaviness, followed by dysphagia, limited tongue movement, episodic involuntary jaw closure resembling trismus, and involuntary tongue biting during attempted tongue protrusion. He denied dyspnea, choking on liquids, limb numbness, weakness, or other motor deficits. Brain magnetic resonance imaging (MRI), magnetic resonance angiography, and diffusion-weighted imaging performed at an outside hospital showed only a few white matter hyperintensities, and laryngoscopy suggested a possible epiglottic papilloma. His medical history was unremarkable. Neurological examination on admission revealed mild dysarthria, restricted mouth opening, limited tongue protrusion with slight rightward deviation, flattening of the left forehead wrinkles and nasolabial fold, restricted left gaze in both eyes, and horizontal right-beating nystagmus. Limb strength was preserved, muscle tone was normal, deep tendon reflexes were normoactive, and left lateral lumbosacral tenderness radiated to the medial aspect of the left lower limb. The initial working diagnosis was multiple cranial neuropathy, with possible intracranial infection.

Routine laboratory tests and inflammatory markers were unremarkable, as were serum Lyme antibody testing and blood metagenomic next-generation sequencing (mNGS). Cerebrospinal fluid (CSF) analysis showed opening pressures of 200 mm H_2_O on April 8 and 210 mm H_2_O on April 21, white blood cell counts of 0 and 2 cells/µL, respectively, and mildly elevated protein levels of 477 and 554 mg/L; Gram, acid-fast, and fungal stains were negative. Serum GAD65 antibody was negative.

Brain and head-and-neck imaging revealed no definite structural lesion to explain the clinical presentation, showing only nonspecific abnormalities, including mild cerebral white matter lesions, paranasal sinus mucosal changes, tongue-base enhancement of uncertain etiology, mild sublingual gland swelling and enhancement, right epiglottic wall thickening, shallow appearance of the right vallecula and left piriform sinus, and mildly enlarged cervical lymph nodes. Laryngoscopy suggested chronic pharyngitis and possible pharyngeal papilloma. These nonspecific abnormalities initially raised concern for orofacial infection but did not establish an infectious or neoplastic cause.

The combination of dysphagia, throat pain, trismus-like jaw closure, and nonspecific oropharyngeal and maxillofacial abnormalities on imaging initially raised concern for infectious multiple cranial neuropathy, tetanus, or orofacial infection. He received sequential antimicrobial therapy with tinidazole plus levofloxacin, followed by linezolid, metronidazole, and doxycycline after multidisciplinary consultation, together with pregabalin for analgesia and oral diazepam for spasm relief. His symptoms improved only transiently and subsequently worsened, arguing against a purely infectious process.

On May 6, he developed neck, truncal, and proximal left lower-limb stiffness, gait impairment, and perioral cutaneous hypersensitivity. Tapping the facial skin elicited facial muscle twitching, and light touch over the perioral muscles induced marked contraction. Needle electromyography (EMG) performed during attempted relaxation demonstrated continuous synchronous motor unit discharges in the left rectus abdominis and left T10 paraspinal muscles (**Figure 1**), with similar continuous activity recorded bilaterally from the sternocleidomastoid muscles. Repetitive nerve stimulation and limb EMG showed no evidence of neuromuscular junction disorder, neuropathy, or myopathy. Together, these findings supported an SPSD phenotype.

**Figure 1:**
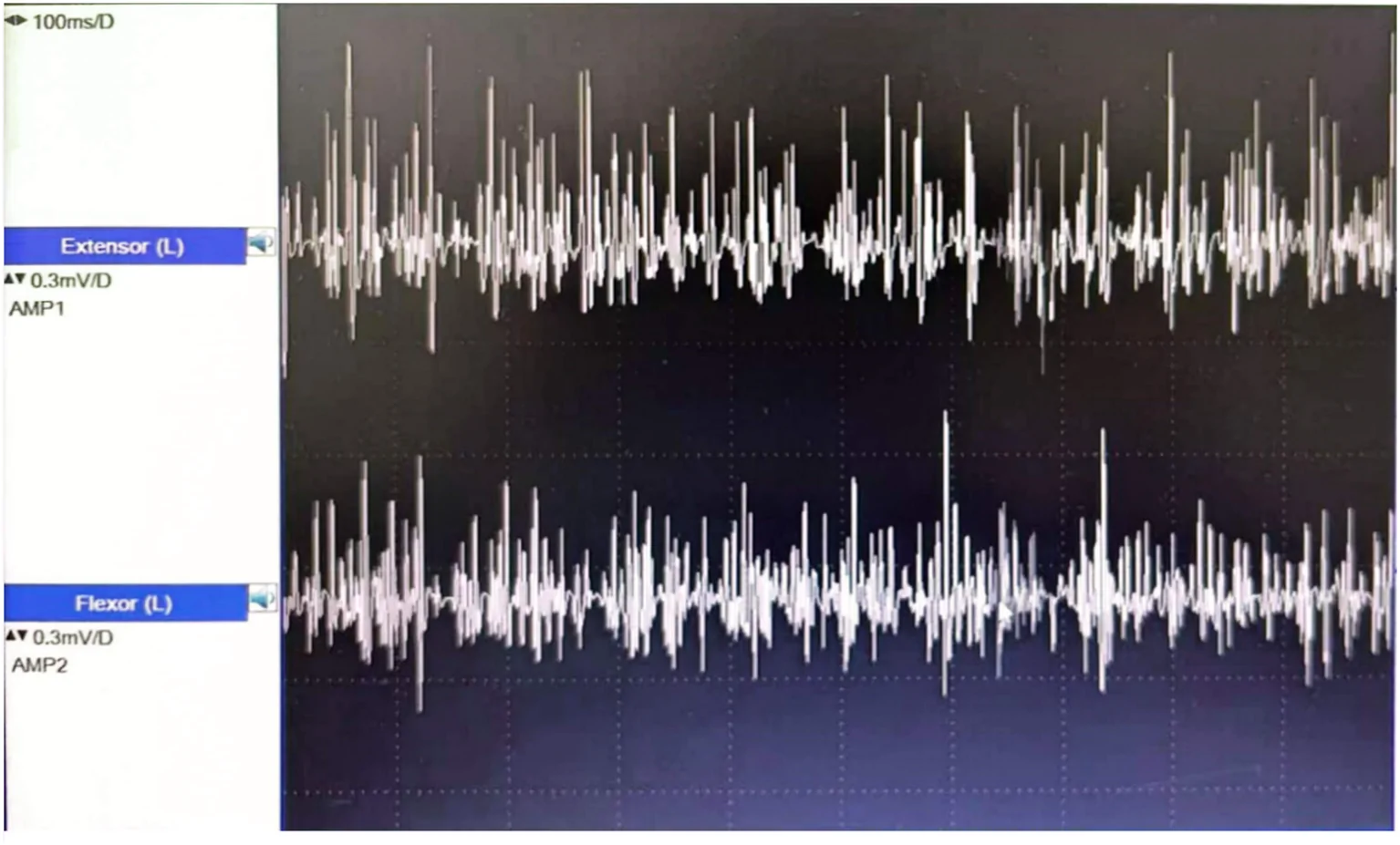
Continuous motor unit activity on needle electromyography. Simultaneous needle EMG recording during attempted relaxation demonstrated persistent continuous motor unit activity in the left T10 paraspinal muscle (upper trace; trunk extensor) and left rectus abdominis muscle (lower trace; trunk flexor), indicating sustained coactivation of antagonistic axial muscles. Sweep speed, 100 ms/division; sensitivity, 0.3 mV/division for both channels.

On May 10, serum testing for SPSD-related antibodies was positive for glycine receptor 1 immunoglobulin G (GlyR1-IgG), with a reported semiquantitative value of 1:100 (**Figure 2**). The initial serum GlyR1-IgG and GAD65-IgG results were obtained using a cell-based indirect immunofluorescence assay (CBA) with transfected mammalian cells. Serum was tested at a fixed 1:10 dilution, and positive results were assigned semiquantitative values by comparing fluorescence intensity with reference standards rather than by serial endpoint titration. GAD65-IgG was negative using this assay. GlyR1-IgG testing was not performed in CSF. The combination of subacute progression, brainstem involvement, stimulus-induced muscle contractions, progressive stiffness, continuous agonist-antagonist motor unit activity, and GlyR1-IgG positivity established the diagnosis of GlyR-Ab-positive PERM. Chest and abdominopelvic computed tomography revealed no radiological evidence of malignancy, although follow-up chest imaging was recommended for nonspecific pulmonary findings.

**Figure 2:**
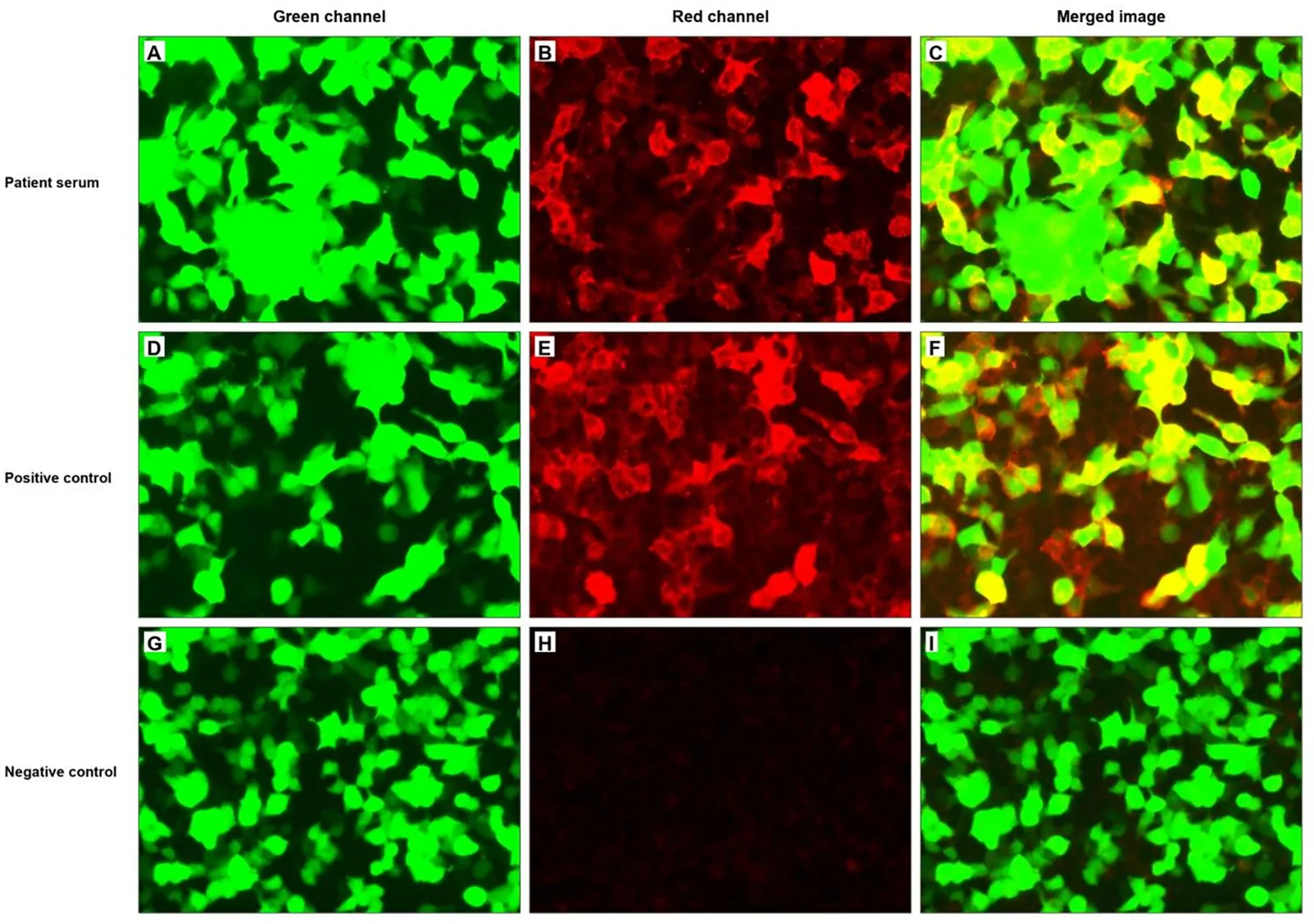
Serum GlyR1-IgG antibody testing by cell-based indirect immunofluorescence assay. **(A–C)** patient serum; **(D–F)** positive control; **(G–I)** negative control. **(A, D, G)** show the green channel indicating GlyR1 expression; **(B, E, H)** show the red channel indicating human IgG binding; **(C, F, I)** show merged images. Patient serum showed GlyR1-IgG positivity with a reported semiquantitative value of 1:100.

On May 10, high-dose intravenous methylprednisolone 500 mg/day was initiated together with intravenous efgartigimod as adjunctive FcRn-targeted therapy. At treatment initiation, the patient weighed 45 kg and was 156 cm tall (body mass index, 18.5 kg/m²). The intended weight-based dose of 10 mg/kg corresponded to 450 mg; because efgartigimod was supplied in 400-mg single-use vials, the dose was rounded down to 400 mg (approximately 8.9 mg/kg) per infusion to avoid opening a second vial for the remaining 50 mg. Efgartigimod was administered once weekly for four consecutive weeks. Methylprednisolone was tapered to 240 mg/day on May 13 and 120 mg/day on May 16. On May 19, treatment was switched to oral prednisone 60 mg/day, with mycophenolate mofetil 500 mg twice daily and diazepam for symptomatic control. Dysphagia, orofacial symptoms, and gait impairment improved markedly, and the patient was discharged in stable condition on May 30. At his most recent outpatient follow-up on July 15, 2026, approximately 14 months after discharge, he remained asymptomatic; diplopia, facial sensory disturbances, restricted mouth opening, and limb stiffness had resolved, and neurological examination was unremarkable. At the 14-month follow-up, serum GlyR1-IgG remained positive, with a reported titer of 1:100 on testing performed at a different laboratory using a transfected-cell CBA. Because the initial and follow-up assays used different titration and interpretation protocols, their numerical results were not considered directly comparable. He was advised to continue oral mycophenolate mofetil 500 mg twice daily as maintenance therapy. The diagnostic and therapeutic course is summarized in **Figure 3**.

**Figure 3:**
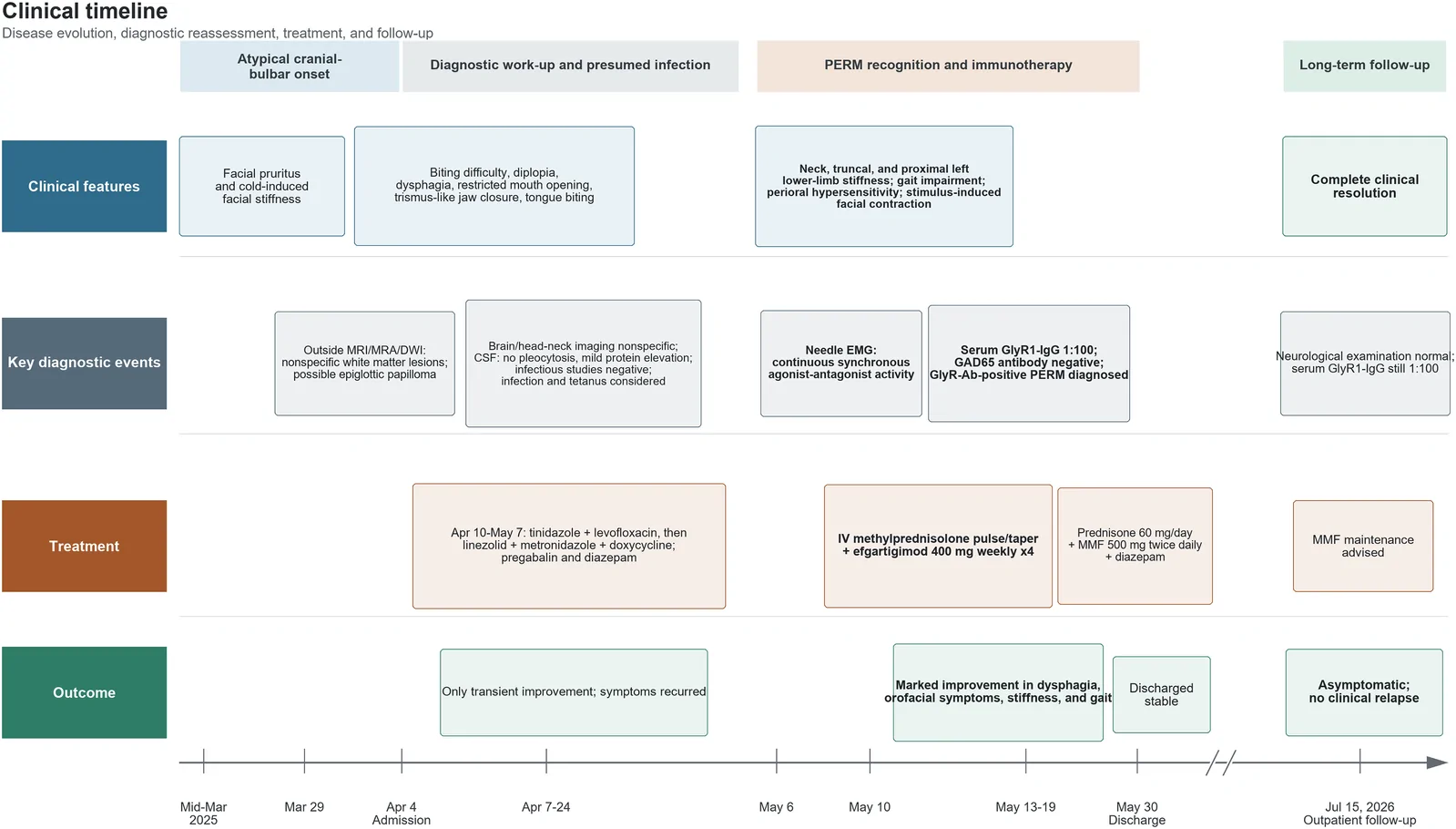
Clinical timeline of disease progression, diagnostic evaluation, treatment, and follow-up. Early cranial-bulbar and orofacial manifestations prompted evaluation for infection, tetanus, and structural lesions. The emergence of axial and limb stiffness and stimulus-induced facial contractions, together with continuous agonist-antagonist motor unit activity and serum GlyR1-IgG positivity, established the diagnosis of GlyR-Ab-positive PERM. Treatment comprised high-dose intravenous methylprednisolone and four weekly doses of efgartigimod, followed by oral prednisone and mycophenolate mofetil, with diazepam for symptomatic control. At follow-up on July 15, 2026, the patient was asymptomatic despite persistent serum GlyR1-IgG positivity at a titer of 1:100. CSF, cerebrospinal fluid; EMG, electromyography; GAD65, glutamic acid decarboxylase 65; GlyR1-IgG, glycine receptor 1 immunoglobulin G; MMF, mycophenolate mofetil; PERM, progressive encephalomyelitis with rigidity and myoclonus.

## Discussion

### Clinical heterogeneity of SPSD and antibody-associated phenotypes

**Table 1** and **Supplementary Table S1** summarize the major SPSD phenotypes and their antibody-associated patterns; therefore, only features most relevant to the present case are discussed here. SPSD is clinically heterogeneous, with overlapping phenotypes ranging from classic SPS and partial SPS to SPS-plus, paraneoplastic SPS, GAD65-associated cerebellar ataxia, and PERM (1, 3). Although these phenotypes may overlap, differences in disease tempo, anatomical distribution, associated neurological features, tumor association, and antibody profile can guide diagnostic classification and therapeutic decision-making (10, 11).

**Table 1:** Clinical-immunological features and treatment considerations for PERM and paraneoplastic SPS.

SPSD phenotype	Typical clinical features	Common antibody profile	Therapeutic focus	Prognosis
PERM	Severe SPSD phenotype with rapidly progressive rigidity, painful spasms, myoclonus, hyperekplexia, brainstem signs, dysphagia, trismus, ophthalmoparesis, autonomic dysfunction, and possible respiratory failure (2).	GlyR-IgG is highly characteristic; GAD65-IgG, amphiphysin-IgG, DPPX-IgG, GABA-A receptor antibodies, or multiple paraneoplastic antibodies may occur (2, 4).	Early aggressive immunotherapy is emphasized, often with corticosteroids, IVIG, plasma exchange, and maintenance immunosuppression; ICU support and tumor treatment may be required (1, 3, 9).	Potentially life-threatening but often immunotherapy responsive, especially in GlyR-IgG-positive disease; relapse may occur and maintenance therapy may be needed (2).
Paraneoplastic SPS	SPS or stiff-limb phenotype with atypical or subacute progression; neck and upper-limb stiffness may be prominent, with possible encephalopathy, myelopathy, neuropathy, or cerebellar features (11, 21).	Amphiphysin-IgG is most characteristic; gephyrin-IgG, GlyR-IgG, Ri-IgG, GAD65-IgG, or other paraneoplastic antibodies may occur (21).	Tumor screening and oncologic treatment are essential; immunotherapy may include corticosteroids, intravenous immunoglobulin (IVIG), plasma exchange, rituximab, or cyclophosphamide in selected severe cases (22).	Depends on tumor control, neurological severity, and treatment timing; neurological improvement may follow tumor treatment but can be incomplete (21).

DPPX, dipeptidyl peptidase-like protein 6; GABA-A, gamma-aminobutyric acid type A; GAD65, glutamic acid decarboxylase 65; GlyR, glycine receptor; ICU, intensive care unit; IgG, immunoglobulin G; IVIG, intravenous immunoglobulin; PERM, progressive encephalomyelitis with rigidity and myoclonus; SPS, stiff-person syndrome; SPSD, stiff-person spectrum disorders.

In the present patient, the central diagnostic challenge was recognizing that an initially localized cranial-bulbar and orofacial presentation later evolved into a GlyR-Ab-positive PERM phenotype. In general, GAD65-IgG is most often associated with classic SPS and cerebellar ataxia, amphiphysin-IgG with paraneoplastic SPS, and GlyR-IgG with PERM or SPS-plus presentations, although substantial clinical overlap exists (2, 12). Treatment should be individualized according to disease severity, antibody profile, relapse risk, and malignancy status, with symptomatic GABAergic therapy combined with immunotherapy when appropriate (1, 3).

### Early presentations and diagnostic pitfalls

Brainstem-predominant onset is a recognized presentation of GlyR-Ab-positive PERM. In the 41-patient series reported by Guasp et al., the predominant initial manifestations were brainstem symptoms, mainly dysphagia and trismus, in 23 patients (56%), and dysesthesias or pruritus in 7 patients (17%) (2). Therefore, bulbar symptoms and pruritus should not individually be regarded as novel manifestations. The present case illustrates how these recognized features may remain diagnostically misleading when they initially occur without objective axial or limb rigidity and are accompanied by nonspecific oropharyngeal imaging abnormalities.

Previous individual reports provide useful comparisons. Irie et al. described unilateral facial stiffness and trismus followed within days by lower-limb rigidity and myoclonus, demonstrating a similar progression from localized cranial symptoms to more widespread motor involvement (13). Yao et al. emphasized inspiratory laryngeal stridor as a prominent manifestation (6), whereas Jiang et al. described delayed recognition after an initial diagnosis of anxiety disorder (8). In our patient, facial pruritus, restricted mouth opening, involuntary jaw closure, diplopia, and dysphagia initially prompted evaluation for infectious cranial neuropathy, tetanus, and orofacial infection. The subsequent emergence of axial and limb stiffness and stimulus-induced facial contractions, together with continuous motor unit activity on EMG, prompted diagnostic reconsideration. Comparison with these reports emphasizes the value of reassessing the diagnosis as symptoms evolve and obtaining targeted antibody testing when cranial-bulbar symptoms are accompanied by features of motor hyperexcitability.

### Diagnostic clues and antibody-guided treatment in PERM

PERM represents the most severe end of the stiff-person spectrum disorders and is characterized by subacute or rapidly progressive rigidity, painful spasms, myoclonus, hyperekplexia, brainstem dysfunction, long-tract signs, and autonomic instability (2). Ancillary tests are supportive but not independently diagnostic, as brain MRI, spinal MRI, and CSF findings may be normal or nonspecific (2, 7). Needle or surface EMG may provide an important diagnostic clue by demonstrating continuous motor unit activity or agonist-antagonist co-contraction, although EMG can be difficult to perform or inconclusive in some severely affected patients (2, 14). Therefore, when PERM is clinically suspected, early serum and CSF testing for SPSD-related antibodies, including GlyR, GAD65, amphiphysin, DPPX, and relevant paraneoplastic antibodies, is essential (2, 14).

Antibody status may inform both clinical interpretation and therapeutic strategy. Because GlyR is a cell-surface inhibitory synaptic target, GlyR-Ab-positive PERM is generally considered more directly antibody mediated and often responds to immunotherapy, including corticosteroids, IVIG, plasma exchange, and maintenance immunosuppression when relapse risk is high (2, 7, 15). By contrast, GAD65-associated PERM may overlap with classic SPS, SPS-plus, cerebellar ataxia, epilepsy, or limbic encephalitis, and immunotherapy responses may be less predictable, particularly when cerebellar ataxia is prominent (16). DPPX-associated PERM should be considered when rigidity and myoclonus are accompanied by neuropsychiatric symptoms, cognitive decline, weight loss, gastrointestinal or urinary dysautonomia, allodynia, cerebellar ataxia, or marked central nervous system hyperexcitability; severe or relapsing cases may require B-cell-directed therapy (14). Paraneoplastic or multiple-antibody PERM warrants systematic tumor screening and repeated surveillance, with treatment combining neurological immunotherapy and tumor-directed therapy when a malignancy is identified (17, 18). Although systemic malignancy screening with chest and abdominopelvic CT was negative in this patient, continued age-appropriate surveillance remains advisable because an occult or subsequently emerging malignancy cannot be completely excluded. Antibody-negative PERM remains a clinical diagnosis after exclusion of mimics and may still respond to immunotherapy, including plasma exchange in refractory or relapsing cases (9). In this patient, GlyR1-IgG positivity, GAD65 antibody negativity, a brainstem-predominant onset, and stimulus-induced motor phenomena were consistent with GlyR-Ab-positive PERM, with the favorable response to immunotherapy providing additional clinical support. Overall, PERM treatment should be individualized according to disease tempo, respiratory or autonomic severity, antibody status, malignancy status, and relapse risk.

### Treatment considerations

Efgartigimod reduces circulating IgG by inhibiting neonatal Fc receptor (FcRn)-mediated IgG recycling, providing a mechanistic rationale for its consideration as an adjunctive treatment in antibody-mediated neurological disease (19). Clinical improvement has been reported in three patients with anti-GAD65-positive SPS and coexisting myasthenia gravis, but these observations cannot be directly extrapolated to GlyR-Ab-positive PERM (20). In the present patient, efgartigimod was initiated together with high-dose corticosteroids, with symptomatic treatment and subsequent maintenance immunosuppression. Its independent contribution to improvement cannot therefore be determined, and this case does not establish its efficacy in PERM. Its potential adjunctive role requires further evaluation.

This report has several limitations. Its single-case design and the use of multimodal therapy, including high-dose corticosteroids, efgartigimod, diazepam, and subsequent mycophenolate mofetil, preclude determination of the independent contribution of any individual treatment. Standardized stiffness or disability scales were not prospectively applied. In addition, GlyR1-IgG was assessed only in serum; CSF antibody testing was not performed, precluding evaluation of serum–CSF concordance and possible intrathecal antibody production. Although repeat serum testing approximately 14 months after discharge demonstrated persistent GlyR1-IgG positivity, the initial and follow-up samples were analyzed by different laboratories using different CBA titration and interpretation protocols. The initial result was based on semiquantitative comparison of fluorescence intensity at a fixed serum dilution, whereas the follow-up result was based on dilution-dependent endpoint assessment. Therefore, the identical reported values of 1:100 cannot be interpreted as a true biological titer plateau or used to assess longitudinal antibody dynamics. Previous work found a tendency toward higher GlyR-Ab titers in patients with PERM or brainstem encephalitis, but no clear relationship between antibody titer and clinical phenotype was identified (4). However, because GlyR-Ab titers were not measured serially during treatment and total IgG levels were unavailable in our patient, the relationships between antibody dynamics, disease severity, and treatment response could not be assessed. Further studies incorporating standardized clinical assessments, longitudinal antibody testing with consistent assay methods, total IgG measurements, and systematic safety monitoring are needed to characterize treatment responses and determine the contributions of individual therapies in GlyR-Ab-positive PERM.

## Patient perspective

Following treatment, the patient experienced progressive improvement in dysphagia, facial discomfort and stiffness, and gait difficulty. At the most recent follow-up, he reported complete resolution of these symptoms, with no remaining limitations in oral intake or ambulation and no recurrence of diplopia or limb stiffness.

## Data Availability

The raw data supporting the conclusions of this article will be made available by the authors, without undue reservation.
