# Supplementary material for: Facial pruritus and stiffness as the initial manifestations of glycine receptor antibody-positive progressive encephalomyelitis with rigidity and myoclonus: a case report and literature review
Source: Front Immunol. 2026 Oct 9;17:1955606. doi: 10.3389/fimmu.2026.1955606 (PMC13690751; doi:10.3389/fimmu.2026.1955606)
Supplement: Supplementary file 1 [file Table1.docx]

Supplementary Table S1. Additional clinical-immunological phenotypes and treatment considerations across the stiff-person spectrum disorders

| **SPSD phenotype** | **Typical clinical features** | **Common antibody profile** | **Therapeutic focus** | **Prognosis** |
| --- | --- | --- | --- | --- |
| Classic SPS | Axial and proximal limb stiffness, painful stimulus-sensitive spasms, hyperlordosis, exaggerated startle, gait impairment, falls, and anxiety or task-specific phobia.(15) | High-titer GAD65-IgG is most typical; CSF GAD65-IgG supports the diagnosis; GlyR-IgG and amphiphysin-IgG are uncommon.(16) | GABAergic symptomatic therapy, mainly benzodiazepines and baclofen; IVIG is the best-supported first-line immunotherapy; rituximab or plasma exchange may be considered in refractory disease. (1, 3, 12) | Usually chronic and disabling but often treatment responsive; delayed diagnosis may result in persistent gait impairment and functional disability. (15) |
| SPS-plus | Classic SPS features with additional neurological involvement, especially cerebellar or brainstem dysfunction, abnormal eye movements, dysarthria, ataxia, epilepsy, myoclonus, or cognitive symptoms. (13, 16) | Often GAD65-IgG positive; GlyR-IgG, DPPX-IgG, gephyrin-IgG, or other antibodies may occur according to phenotype. (13, 16) | Often requires more intensive immunotherapy than classic SPS; IVIG, corticosteroids, rituximab, or plasma exchange may be selected according to severity and response. (1, 3) | More heterogeneous than classic SPS; brainstem or cerebellar involvement is associated with diagnostic delay, misdiagnosis, and potentially worse outcome. (13, 16) |
| Partial SPS / stiff-limb syndrome | Focal or segmental stiffness and spasms, usually beginning in one lower limb; axial rigidity and hyperlordosis may be absent early, and some cases later generalize. (13) | GAD65-IgG may be present but seropositivity can be limited; amphiphysin-IgG should be considered in atypical or paraneoplastic presentations. (13) | Symptomatic treatment for focal stiffness; IVIG or other immunotherapy when autoimmune SPSD is supported; tumor screening if amphiphysin-IgG or atypical features are present. (1, 3) | Variable; some patients remain focal, whereas others progress to classic SPS; persistent focal disability may occur. (13) |
| GAD65-associated cerebellar ataxia | Gait ataxia, limb or axial ataxia, dysarthria, diplopia, central vertigo, nystagmus, and other oculomotor abnormalities; may occur alone or overlap with SPSD. (14, 19, 20) | High-titer serum GAD65-IgG is typical; CSF GAD65-IgG and CSF-restricted oligoclonal bands may support intrathecal immune activation. (19) | Immunotherapy may include IVIG, corticosteroids, rituximab, or plasma exchange; gait and balance rehabilitation are important. (19) | Often disabling and may respond less completely than classic SPS; cerebellar ataxia and very high GAD65 titers have been associated with poorer outcome. (19) |

*Abbreviations: CSF, cerebrospinal fluid; DPPX, dipeptidyl peptidase-like protein 6; GABA, gamma-aminobutyric acid; GAD65, glutamic acid decarboxylase 65; GlyR, glycine receptor; IgG, immunoglobulin G; IVIG, intravenous immunoglobulin; SPS, stiff-person syndrome; SPSD, stiff-person spectrum disorders. References correspond to the reference list in the main manuscript.*
